# Public awareness of cancer risk factors in the Moroccan population: a population-based cross-sectional study

**DOI:** 10.1186/1471-2407-14-695

**Published:** 2014-09-23

**Authors:** Karima El Rhazi, Bahia Bennani, Samira El Fakir, Ahmadou Boly, Rachid Bekkali, Ahmed Zidouh, Chakib Nejjari

**Affiliations:** Department of Epidemiology and Public Health, Faculty of Medicine and pharmacy of Fez, Sidi Mohamed Ben Abdillah University, B.P 1893, Route Sidi Harazem, Km 2.2, Fez, Morocco; Department of Microbiology and Molecular Biology, Team of Microorganisms and Oncogene factors Faculty of Medicine and Pharmacy of Fez, Sidi Mohamed Ben Abdillah University, Fez, Morocco; Lalla Salma Fondation of Prevention and Traitement of Cancers, Rabat, Morocco

**Keywords:** Awareness, Determinants, Cancer, Risk factors, Morocco

## Abstract

**Background:**

In Morocco, knowledge of cancer risk factors, a crucial element in the process of behavioral change, has never been evaluated. This study aims to provide information on the level of awareness of cancer risk factors among the Moroccan general population.

**Methods:**

A cross sectional survey was carried out in May 2008, using a stratified sampling method in a representative sample of the Moroccan adult population. The used questionnaire included social and demographic data as well as questions about 14 cancer related factors regarding passive or active smoking, alcoholic beverages, obesity, physical inactivity, food coloring, red meat, fat, salt, fruit, vegetables, olive oil, green tea, coffee, breast-feeding. Subjects had to choose between 3 propositions for each proposed factor (risk factor/Protective factor/Don’t Know). The knowledge score was calculated by summing the correct answer for each proposed factor except coffee and food coloring. The answer was assigned 1 if it’s correct or 0 if it was incorrect or the participant responded ‘don’t know. The maximum knowledge score was 12. Multivariate linear regression model was used to evaluate the determinants of knowledge score.

**Results:**

Among 2891 subjects who participated to the survey, 49.5% were men and 42% were from a rural area. The mean age was 41.6 ± 15.2 years. The mean knowledge score of cancer related factors was 8.45 ± 3.10 points. Knowledge score increased with educational level (β = −0.65 if school year ≤6 versus >6) and housing category (β = 1.80 in high standing housing vs rural housing). It was also higher in urban area, among never smokers and among people never consuming alcohol compared to others groups.

**Conclusion:**

These results provide valuable information necessary to establish relevant cancer prevention strategies in Morocco aiming to enhance and improve people’s knowledge about risk factors especially in some target groups.

## Background

Cancer development is associated with several factors. Since the study by Doll and Peto [1], which made a detailed assessment of various cancers related risks, several epidemiological studies have identified factors which show a causal relationship with cancer development. It has been estimated by various authorities that about one-third of cancers, in western high-income societies, are due to factors related to food and physical activity [2]. As recommended by “World Cancer Research Fund/American Institute for Cancer Research” [2], regular consumption of vegetables, daily physical activity, limited intake of red meat and alcoholic beverages, decrease the risk of cancer development. Therefore, the cancer prevention is possible by behavioral change. This justifies the implementation of preventive actions [3–9]. However, to ensure the effectiveness of such initiatives, the first step consists in understanding the concerns and beliefs of the target population. Indeed, awareness campaigns are crucial in cancer prevention programs. Moreover, knowledge of cancer risk factors is a determinant element in the process of behavioral change [1, 2].

In Morocco, the national annual incidence of cancer is estimated between 30 000 and 40 000 new cases. The most common cancers in Morocco are breast cancer, lung cancer, cervix cancer, colorectal cancer and prostate cancer [10]. Cancer is still a major public health problem because the diagnosis is often delayed and treatment at diagnosed stage is difficult to set up and very expensive [4]. Statistical cancer studies are based on data reported by cancer registry in a given geographical area. Those studies improve epidemiology cancer knowledge in the concerned area. However, knowledge of practices and risk factors associated to cancer has never been evaluated in Morocco. To promote cancer prevention programs, data on the level of knowledge among the target population are needed. Therefore, we conducted a survey on cancer risk factors knowledge in a representative sample of the Moroccan population. This study aims to provide information on the awareness on cancer, among Moroccan general population, regarding some risk factors.

## Methods

### Sampling design

A cross sectional survey was carried out in May 2008, using a stratified two-stage sampling method, on a national random sample of the Moroccan population aged 18 years and above. Sample size was calculated to represent the general population on the basis of 15% risk factor prevalence, 2% precision, 95% CI and a cluster effect of 2. Thus, sample size was estimated at 2448 and rounded to 3000 persons to compensate for people refusing to take part or being absent during the survey. The people to be surveyed were selected at random from 150 communes, in clusters of twenty households per commune. A cluster was defined as a neighbourhood in an urban area and a locality in a rural area. One cluster was selected at random from each commune included in the survey and one person aged 20 years or above from each household of the cluster was selected at random. The total cluster selection was done proportionally to the distribution of the Moroccan population in urban and rural areas (53 and 47%, respectively) [11]. The details of the numbers of included communes and therefore of included clusters by origin (urban or rural) in each named region of Morocco are given in the Table 1.

**Table 1:** **Repartition of communes included in the study by origin (urban/rural) in each region of Morocco**

	Urban	Rural	Total
Sahara*	3	1	4
Souss-Massa-Draa	7	8	15
Gharb-Chrarda-Beni Hssen	4	4	9
Chaouia-Ouardigha	4	4	8
Marrakech-Tensift-Al Haouz	7	8	15
Oriental	6	3	10
Grand Casablanca	19	2	20
Rabat-Salé-Zemmour-Zaër	11	2	13
Doukkala-Abda	4	6	9
Tadla-Azilal	3	4	7
Meknès-Tafilalet	7	4	11
Fès-Boulemane	6	2	8
Taza-Al Hoceima-Taounate	2	6	8
Tanger-Tétouan	8	5	13
Total	91	59	150

*Sahara = Oued Eddahab-Lagouira, Laâyoune-Boujdour-Sakia-Lhamra, GuelmimEs-Smara.

Ethical approval was applicable to the present study under the guidelines in use for epidemiologic studies and which comply with the declaration of Helsinki. It was approved by the ethics committee of Fez University Hospital Center. All subjects gave their consent before answering the survey.

### Knowledge level variables

The questionnaire of this survey contained questions on the awareness of various cancer risk factors according to international literature. Therefore, 14 cancer presumed related factors were studied, including passive and active smoking, alcoholic beverages, obesity, physical inactivity, food coloring, red meat, fat, salt, fruit, vegetables, olive oil, green tea, coffee, breast-feeding. Most included items were chosen based on: i) their potential link as risk or protective factor for some type of cancers as described elsewhere [2, 12–14], ii) included food items (food coloring, red meat, fat, salt, fruit, vegetables, olive oil, green tea, coffee, breast-feeding) are commonly used in Moroccan population, iii) included attitudes items (passive and active smoking, alcoholic beverages, obesity, physical inactivity) are frequently adopted in Morocco. Questionnaire about the knowledge of these items is given in Table 2.

**Table 2:** **Questionnaire about Risk or Protector factor Knowledge of cancer in Moroccan Population**

A votre connaissance, les éléments suivants constituent t-ils un facteur de risque ou facteur protecteur de cancer?			
bcb	**Facteur de risque**	**Facteur protecteur**	**Ne sait pas**
Tabac actif	□	□	□
Tabac passif	□	□	□
Alcool	□	□	□
Obésité	□	□	□
Sédentarité	□	□	□
Viandes rouges	□	□	□
Consommation excessive de graisses	□	□	□
Consommation excessive de sel	□	□	□
Fruit	□	□	□
Légumes	□	□	□
Thé vert	□	□	□
Café	□	□	□
Huile d’olive	□	□	□
Allaitement maternel	□	□	□
Colorants alimentaires	□	□	□

For each candidate cancer risk factor, three answers were proposed: 1/ it is a risk factor, 2/ it’s a protective factor 3/ don’t know. The people’s knowledge of cancer risk factors was assessed by choosing the correct answer among these three propositions for each of the proposed factors. Each answer was scored 1 if it was correct or 0 if it was incorrect or the participant responded ‘don’t know’. For smoking item, passive and active smoking which concern the same risk factor, were accounted as one item. The answer was correct if the answer of passive and/or active smoking was correct and incorrect if not. Coffee and food coloring were not considered when calculating the knowledge score because of the controversial results on their cancer link. Then, total knowledge score ranged from 0 (the subject did not recognize any factor) to 12.

### Independent variables

Data concerning socio demographic factors (age, gender, region of residence, educational level, marital status, employment status, average family income, self-reported health status, family history of cancer, physical activity, smoking and alcohol attitudes were also collected.

The questionnaire was developed by the authors and was stated in French which is the second Moroccan state language. It was administered in local dialect by trained pair (one man and one woman) including physicians and nurses chosen from the same regions as the participants. The data were collected in the subjects’ homes during a personal interview which was carried out homogeneously from Monday to Sunday. The questionnaire’s face validity was checked in a pilot study in 20 participants and showed that the questionnaire was acceptable and understandable. All information collected on individuals has been kept confidential and anonymous.

### Statistical analysis

All data statistical analyses were conducted using SPSS 17.0 software. Knowledge level score and related socio demographic variables were analyzed using the Student t- test or one-way analysis of variance. Variables with P ≤ 0.20 on univariate analysis were entered in multivariate linear regression model to evaluate the knowledge level score of the cancer risk factors and its determinants.

## Results

### Population characteristics

Among the 3000 persons enrolled in this study, only 2891 (96.5%) took part in the survey. People, who are not included in the survey refused to participate to the survey or were absent (3.5%). This proportion was higher in urban areas (4.9%) than rural areas (1.4%).

Among all participants, 1433 were men (49.5%), 1461 women (50.5%) and 42% were from rural areas. The average age was 41.6 (standard deviation (SD) = 15.2) years and 43.4% were illiterate. The average family income was less than 2000 MAD/month (equivalent currency exchange is: 1 MAD = 0.09 h) for 52.5% of participants. A quarter of participant (25%) and 7.9% were currently or formerly smokers and alcohol consumers respectively. (Table 3).

**Table 3:** **Socio-demographic characteristics of the study participants (n = 3000)**

	N	%
***Origin***		
Rural	1209	41.7
Urban	1687	58.3
Total	2896	100.0
***Age groups (years)***		
< 35	1083	37.5
35 – 49	943	32.6
> = 50	865	29.9
Total	2891	100.0
***Gender***		
Male	1433	49.5
Female	1463	50.5
Total	2896	100.0
***Marital status***		
Married	1986	69.2
Single or divorced or widowed	886	30.8
Total	2872	100.0
***Educational level***		
Illiterate	1250	43.5
< 6 years school	838	29.1
≥ 6 years school	788	27.4
Total	2876	100.0
***Occupational activity***		
Active or student	1367	47.2
Retired or unemployed	385	13.4
Housewife	1113	38.8
Total	2865	100.0
***Average family income***		
< 2000	1456	60.2
2 000–4 999	714	29.5
≥ 5000	248	10.3
Total	2418	100.0
***Housing category***		
Luxurious or modern	450	15.5
New medina	416	14.4
Old medina	546	18.9
Poor housing or slums	303	10.5
Rural housing	1181	40.8
Total	2896	100.0
***Tobacco consumption***		
Current smokers	441	15.9
Ex smokers	252	9.1
Never smokers	2088	75.1
Total	2781	100.0
***Alcohol consumption***		
Current consumers	96	3.4
Ex consumers	128	4.5
Never consumers	2605	92.1
Total	2829	100.0
***Physical activity***		
Yes	1610	55.6
No	1025	35.4
Total	2635	100.0
**Family history of cancer**		
Yes	380	13.6
No	2404	86.4
Total	2784	100.0
**Health problem**		
Yes	957	33.1
No	1936	66.9
Total	2893	100.0

Among all participants, only 2.2% showed a correct knowledge about all mentioned risk factors. For factors considered as cancer risk factors, 90.8% and 86.3% had correct knowledge about active and passive smoking respectively, 81.0% for alcohol consumption, 62.7% for obesity, 61.3% for fat, 59.9% for physical inactivity, 59.2% for salt and only 36.9% for red meat. For other factors considered as protecting factors, 88.9% of participants give a correct answer for olive oil, 86.4% for vegetables, 83.4% for fruit, 73.1% for breast feeding and 65.4% for green tea. For controversial factors, 17.6% and 40.0% of participants believe that food coloring and coffee are risk factors respectively.

The average knowledge score of cancer risk factors in participants was 8.5 ± 3.1 points. Significant differences in average knowledge score were observed depending on the area of origin, level of education, housing category and family income. Hence, subjects from urban area, who spent more 6 years at school, with family incomes greater than 5000 MAD and who were living in modern housing had significantly higher knowledge score regarding cancer risk factors (*P* < 0.001, *P* < 0.001, *P* = 0.01 and *P* < 0.001 respectively). Some attitudes were also related to high level of knowledge score, especially tobacco (p = 0.01) and alcohol consumption (p = 0.06). However, gender, marital status, profession and physical activity did not show significant influence on the knowledge score of cancer risk factors (Table 4).

**Table 4:** **Knowledge score of cancer risk factor according to the main demographic and socio-economic characteristics**

	N	Means	SD	p-value
***Origin***				
Rural	1173	8.2	3.3	<0.001
Urban	1644	8.6	2.9	
Total	2817			
***Age groups (years)***				
< 35	1058	8.4	3.1	
35 – 49	919	8.6	3.0	0.30
> = 50	835	8.4	3.2	
Total	2812			
***Gender***				
Male	1379	8.5	2.9	
Female	1438	8.4	3.3	0.53
Total	2817			
***Marital status***				
Married	1926	8.4	3.2	
Single or divorced or widowed	867	8.6	3.0	0.16
Total	2793			
***Educational level***				
Illiterate	1210	8.0	3.4	
< 6 years school	817	8.5	3.0	<0.001
≥ 6 years school	771	9.1	2.5	
Total	2798			
***Occupational activity***				
Active or student	1319	8.5	2.9	
Retired or unemployed	372	8.6	2.9	
Housewife	1095	8.3	3.3	0.17
Total	2786			
***Average family income***				
< 2000	1405	8.3	3.3	
2 000–4 999	700	8.5	2.9	0.010
≥ 5000	242	8.9	2.4	
Total	2347			
***Housingcategory***				
Luxurious or modern	438	9.2	2.4	
New medina	404	8.2	3.2	
Old medina	541	9.1	2.6	
Poor housing or slums	286	7.7	3.2	<0.001
Rural housing	1148	8.2	3.3	
Total	2817			
***Tobacco consumption***				
Current smokers	429	8.3	2.8	
Ex smokers	246	8.3	3.1	0.001
Never smokers	2030	8.8	2.9	
Total	2705			
***Alcohol consumption***				
Current consumers	92	7.7	3.1	
Ex consumers	126	8.4	2.9	0.06
Never consumers	2535	8.4	3.1	
Total	2753			
***Physical activity***				
Yes	1570	8.4	3.1	
No	992	8.6	3.1	0.29
Total	2562			
**Family history of cancer**				
Yes	370	8.4	3.1	0.78
No	2445	8.5	3.1	
Total	2815			
**Health problem**				
Yes	935	8.3	3.1	
No	1880	8.5	3.1	0.11
Total	2815			

Sample of the adult Moroccan population, 2008.

After adjusting for confounding factors, knowledge score increased with educational level and housing category. It was also higher in urban area, among never smokers, people never consuming alcohol and among people without past history of health problem compared to others groups (Table 5).

**Table 5:** **Correlates of knowledge level score of cancer risk factors among the Moroccan population: multivariate analysis**

	Beta	95% CI limits Beta		p-value
***Alcohol consumption***				**0.006**
Never consumers	Reference			
Ex consumers	−1.01	−1.63	−0.39	0.01
Current consumers	−0.09	−0.62	0.45	0.75
***Tobacco consumption***				**0.03**
Never smokers	Reference			
Ex smokers	−0.48	−0.80	−0.15	0.004
Current smokers	−0.46	−0.84	−0.07	0.021
**Housing category**				**<0.001**
Rural housing	Reference			
Luxurious or modern	1.80	0.66	2.93	0.002
New medina	1.18	0.03	2.33	0.044
Old medina	1.84	0.70	2.98	0.002
Poor housing or slums	0.65	−0.433	1.73	0.240
**School year**				**<0.001**
≥ 6 years school	Reference			
Illiterate	−0.65	−0.95	−0.36	<0.001
< 6 years school	−0.33	−0.63	−0.03	0.030
**Health problem**				**0.040**
Yes	Reference			
No	0.24	0.01	0.48	**0.040**
**Origin**				
Rural	Reference			
Urban	1.22	0.12	2.33	**0.030**

## Discussion

This cross-sectional study allowed determining the knowledge level of cancer risk factors among the Moroccan population. The results of this study show that educational level and housing category were significantly associated with a high knowledge score of cancer risk factors. This can be explained by the fact that health knowledge is depending on the socioeconomic level [15, 16]. Effectively, individuals with high economic standards have easier access to outreach programs which were more easily assimilated by individuals with higher education level. These results are consistent with other previous studies [15–17].

A significant association between the area of origin and knowledge score of cancer risk factors has also been observed. The high knowledge score was noted in urban area as reported in other studies [18–21] and can be explained by the difference in health facilities between the urban and rural areas.

Smoking is a widespread habit in many developing countries, it is found as a risk factor in several malignant tumors [3, 22–25]. Its prevalence in Morocco was 18.0% in both sexes [26, 27] and its consumption was significantly associated with a low score knowledge in the current study. However, 85.9% of participants had a good knowledge of tobacco effect. Anastasiou et al. [19] showed that only 58.4% of patients stated that they were aware of smoking as a risk factor for bladder cancer, versus 94.6%, 91.6% and 92.1% who related this risk factor to chronic obstructive pulmonary disease, heart and vascular problems and to lung cancer, respectively [19]. Some authors proposed recommendations [28] on how comprehensive tobacco control policies that address smoking-related inequalities can be developed. Moreno et al. [29] suggested that strategies such as pricing and taxations, regulation of products and restrictions of advertising will also play a very important role besides awareness in achieving behavioral change.

Alcohol consumption was also significantly associated with a low knowledge score. Although alcohol is a risk factor for many type of cancers [1, 30], public awareness on the harmful effect of alcohol consumption concerns mainly cardiovascular diseases [29–31]. In Morocco, despite a low prevalence of alcohol consumption, awareness of the cancer risks associated to excessive alcohol consumption is needed especially because of the increasing of cancer prevalence and alcohol consumption in the last years in this country.

The main limitation of this study is its cross-sectional design. Responses to this type of cross-sectional survey could be affected by social conditions such as information from the mass media and other sources on diseases and their risk factors. Thus, the results might not necessarily reflect actual public awareness. However, the sample study was representative of the whole Moroccan population since the prevalence of socio-demographic factors was similar to that reported in the last general census of habitat and population in 2004 [11]. Another limitation of the study is related to the questionnaire which did not give people the option of saying that a factor is neither risky nor protective. This is likely to have increased the rate of guessing and giving the more accurate knowledge score. To our knowledge, this is the first study that assesses the level of awareness for some cancer risk or protector factors in Morocco. The calculating score was based only on established risk or protective cancer factors. This score may help us to have an approach for the Moroccan public awareness of cancer risk factors which could be useful in the formulation of public health initiatives for cancer prevention.

## Conclusion

Awareness of cancer risk factors among the Moroccan general population seems to be dominated by some behaviors (active or passive smoking and alcohol consumption rather than dietary factors (red meats). Hence, the people’s knowledge level about cancer risk factors must be enhanced in order to improve cancer prevention. This could be possible by modifying the people’s knowledge about cancer risk factors. The results of the present survey provide precious information that could be used for setting up valuable cancer prevention strategies in Morocco.
